# Atypical IκB proteins – nuclear modulators of NF-κB signaling

**DOI:** 10.1186/1478-811X-11-23

**Published:** 2013-04-11

**Authors:** Marc Schuster, Michaela Annemann, Carlos Plaza-Sirvent, Ingo Schmitz

**Affiliations:** 1 Systems-oriented Immunology and Inflammation Research, Helmholtz Center for Infection Research, Braunschweig, 38124, Germany; 2 Institute for Molecular and Clinical Immunology, Otto-von-Guericke University, Magdeburg, 39120, Germany

**Keywords:** NF-kappaB, Atypical IkappaB proteins, BCL-3, IkappaBNS, IkappaBzeta, IkappaBL, Nuclear NF-kappaB modulation, IkappaB eta, MAIL, NFkBID

## Abstract

Nuclear factor κB (NF-κB) controls a multitude of physiological processes such as cell differentiation, cytokine expression, survival and proliferation. Since NF-κB governs embryogenesis, tissue homeostasis and the functions of innate and adaptive immune cells it represents one of the most important and versatile signaling networks known. Its activity is regulated via the inhibitors of NF-κB signaling, the IκB proteins. Classical IκBs, like the prototypical protein IκBα, sequester NF-κB transcription factors in the cytoplasm by masking of their nuclear localization signals (NLS). Thus, binding of NF-κB to the DNA is inhibited. The accessibility of the NLS is controlled via the degradation of IκBα. Phosphorylation of the conserved serine residues 32 and 36 leads to polyubiquitination and subsequent proteasomal degradation. This process marks the central event of canonical NF-κB activation. Once their NLS is accessible, NF-κB transcription factors translocate into the nucleus, bind to the DNA and regulate the transcription of their respective target genes. Several studies described a distinct group of atypical IκB proteins, referred to as the BCL-3 subfamily. Those atypical IκBs show entirely different sub-cellular localizations, activation kinetics and an unexpected functional diversity. First of all, their interaction with NF-κB transcription factors takes place in the nucleus in contrast to classical IκBs, whose binding to NF-κB predominantly occurs in the cytoplasm. Secondly, atypical IκBs are strongly induced after NF-κB activation, for example by LPS and IL-1β stimulation or triggering of B cell and T cell antigen receptors, but are not degraded in the first place like their conventional relatives. Finally, the interaction of atypical IκBs with DNA-associated NF-κB transcription factors can further enhance or diminish their transcriptional activity. Thus, they do not exclusively act as inhibitors of NF-κB activity. The capacity to modulate NF-κB transcription either positively or negatively, represents their most important and unique mechanistic difference to classical IκBs. Several reports revealed the importance of atypical IκB proteins for immune homeostasis and the severe consequences following their loss of function. This review summarizes insights into the physiological processes regulated by this protein class and the relevance of atypical IκB functioning.

## Review

### NF-κB signaling

NF-κB transcription factors are homo- or hetero dimers composed of two REL proteins, such as p50 and p65 [1]. This family consists of p65/RelA, p50, p52, RelB and c-Rel. Their common structural motif is the Rel homology domain (RHD) [2]. It contains a dimerisation sequence for the interaction with other REL proteins, a nuclear localization signal (NLS) regulating its subcellular localization and a DNA binding motif for the interaction with κB sites in regulatory sequences of their respective target genes. Transactivation domains, TAD, are found in p65/RelA, c-Rel and RelB, whereby NF-κB dimers containing at least one of these subunits can induce transcription [2]. On the other hand, NF-κB homodimers of p50 and p52 function as transcriptional repressors due to the lack of such a sequence [2]. They either compete for activating NF-κB transcription factors by occupation of DNA binding sites, or recruit gene-silencing proteins such as histone deacetylases (HDACs) [3], or inhibit transcription by use of both mechanisms. Each REL-protein subunit, with its individual and slightly different DNA-binding domain, contributes to the total DNA-affinity of the dimeric transcription factor [4-6]. Thus, the optimal sequence for NF-κB binding is not identical among the different dimer combinations. This results in a magnitude of optimal regulatory sequences. The diversity of ideal binding sites, the multitude of κB-sites in the DNA and the existence of suppressive and inducive NF-κB dimers are the reasons of the complexity and versatility of the downstream signaling network.

NF-κB can be activated in two different fashions, called canonical and non-canonical NF-κB activation. Both pathways use a complex formed by IκB kinase proteins, however, in slightly different compositions [2,7]. Regulation of the upstream signaling events and detailed differences between the canonical and non-canonical NF-κB activation were previously illustrated and are not part of this review [2,7].

### Canonical NF-κB activation

The canonical signaling is initiated by a variety of receptors, like members of the TNF receptor super-family, Toll-like receptors, interleukin receptors and antigen receptors of B and T cells [2]. Their common downstream signaling complex is a trimeric IκB kinase complex consisting of the catalytic subunits IKKα, IKKβ and the regulatory subunit IKKγ/NEMO [8,9]. The sequestration of NF-κB in the cytoplasm is mediated by the association of classical IκBs such as the prototypical protein IκBα to inhibit NF-κB binding to the DNA [10-13]. The characteristic structural motif of IκB proteins is a repetitive sequence of 6 to 10 ankyrin domains [2]. Binding of these ankyrin repeats to the REL homology domain of NF-κB results in masking of the NLS [14,15]. Crystallography demonstrated that the ankyrin domain of IκBα localizes between the carboxy-terminal Ig-like sequences of the REL homology domains of two NF-κB subunits [16]. When the NLS is accessible the NF-κB transcription factor can localize in the nucleus and bind to the DNA, which depends on IκBα degradation [17,18]. In case of IκBα this process is initiated by phosphorylation of the serine residues 32 and 36 by activated IKKβ [18-20]. The phosphorylated serines within the so-called “destruction box” of IκBα are subsequently recognized by the E3 ligase βTRCP leading to polyubiquitination and eventually causing proteasomal degradation of IκBα [17,21-23]. As the NLS of the NF-κB dimer is accessible the transcription factor localizes into the nucleus and modulates transcription via binding to the DNA.

### Non-canonical NF-κB activation

The non-canonical NF-κB activation depends on an IKKα homodimer, activated for example by triggering of the BAFF receptor, CD40 or the lymphotoxin-β receptor [7,24,25]. The NF-κB dimers activated in the non-canonical signaling cascade are composed of p52 and RelB [26]. Their NLS sequences are masked intra-molecularly by the precursor protein of p52, p100, which displays carboxy-terminal ankyrin repeats to interact with the REL domains and hide the NLS [27]. Phosphorylation of p100 causes cleavage of the protein into p52 leading to the nuclear translocation of NF-κB [26,28]. As the ankyrin repeats are part of the sequences of p100 as well as p105, the precursor of p50, the existence of a common evolutionary ancestor for both, IκB and REL proteins is reasonable. Alternatively, p100 and p105 could be the result of a gene fusion.

### Nuclear modulation of NF-κB activity

**Figure 1:**
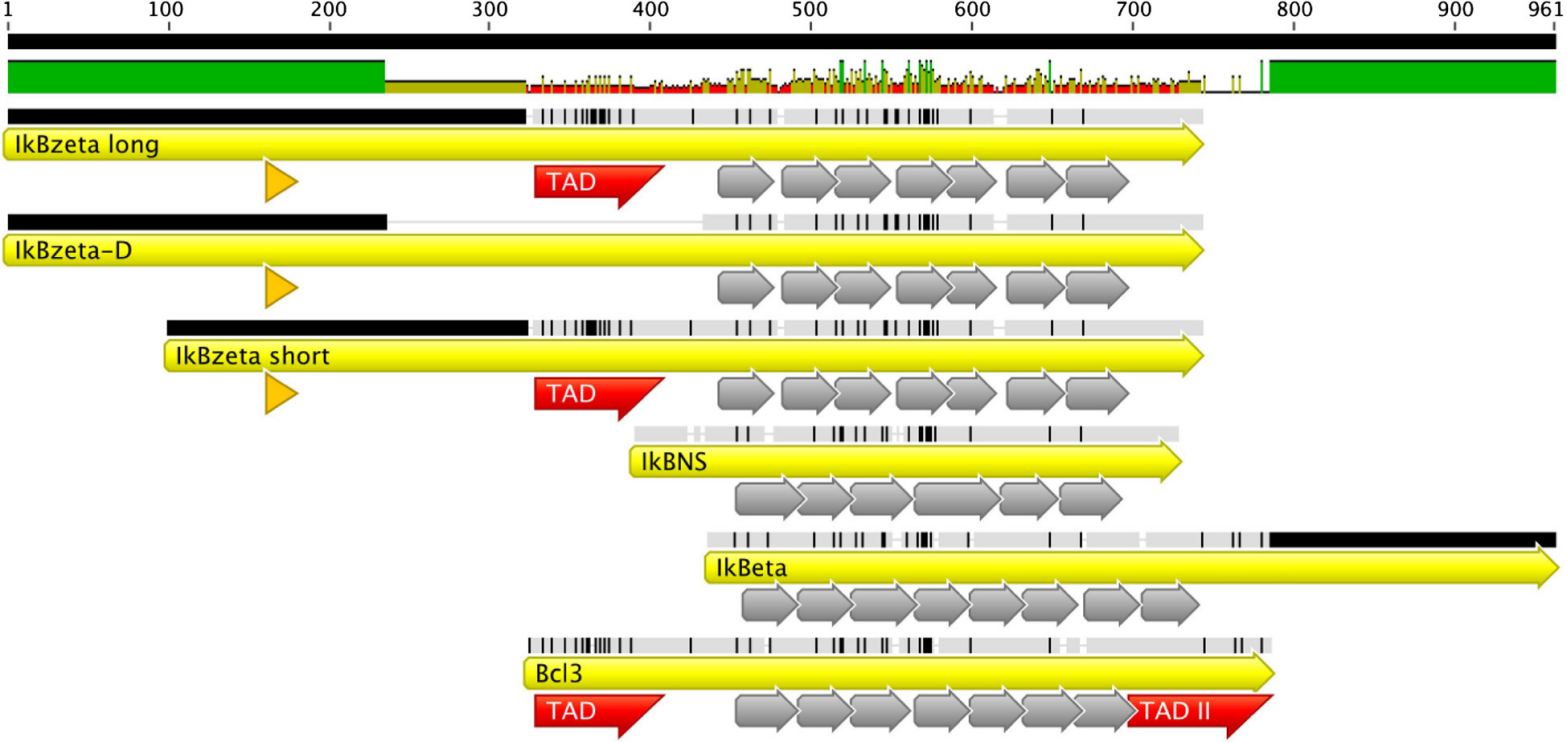
**Alignment of atypical IκB proteins.** Alignment of murine atypical IκB proteins IκBζ long, IκBζD, IκBζ short, IκB_NS_, IκBη and BCL-3 is shown. Position of ankyrin repeats (grey), transactivation domains, TAD, and nuclear localization signal (orange) are indicated within the coding sequence (yellow). Identity between the sequences is indicated in the upper part of the diagram with green showing highest similarity and red lowest similarity. Sequences NP_001152867, NP_082941, NP_085115, NP_291079 and NP_742154 were used for the alignment created by Geneious v5.3 software (Drummond et al., 2010; available at www.geneious.com/).

NF-κB activity is fine-regulated in the nucleus by a variety of mechanisms, including post translational modifications of REL proteins for example sumoylation, phosphorylation, acetylation and ubiquitination [3,29]. Besides, the nuclear IκB proteins of the BCL-3 class BCL-3, IκB_NS_, IκBζ and IκBη can dramatically alter NF-κB-mediated effects via the regulation of dimer exchange, the recruitment of histone modifying enzymes or the stabilization of NF-κB dimers on the DNA. Although, these proteins formally belong to the IκBs due to the presence of ankyrin repeats in their structure (Figure 1), they do not functionally act exclusively as repressors of NF-κB-mediated transcription, but more as NF-κB modulators (Table 1).

**Table 1:** Properties of atypical IκB proteins

	**BCL-3**	**IκBζ**	**IκB**_**NS**_	**IκBη**	**IκBL**
**Alternative names**	B-cell CLL/lymphoma 3 , D19S37, AI528691	Nfkbiz, FLJ30225, FLJ34463, INAP, MAIL, AA408868	Nfkbid, IkB-delta, MGC11314, MGC149503, TA-NFKBH, T-cell activation NFKB-like protein	Ankrd42, FLJ37874, SARP, 4931426M20, 4933417L02Rik	LST1, NF-kappa-B inhibitor-like protein 1
**Chromosomal Localisation (NCBI geneID)**	Human:	Human:	Human:	Human:	Human:
	19q13.1-q13.2	3p12-q12	19q13.12	11q14.1	6p21.3
	(602)	(64332)	(84807)	(338699)	(4795)
	Mouse:	Mouse:	Mouse:	Mouse:	Mouse:
	7A3; 7 9.95 cM	16; 16 C1.2-C1.3	7 B1; 7	7; 7E2	17 B1; 17
	(12051)	(80859)	(243910)	(73845)	18.6 cM (18038)
**Interaction partners**	p50 [35,36,40], p52 [32], c-Jun [41], c-Fos [41], CREB/p300 [41,43], SRC-1 [41], B3BP [42], Tip60 [43], HDAC-1/-3/-6 [37,45]	p50 [64], STAT3 [71], FUSS-DDIT3 [72], p65 [73], RORγ [77], RORα [77]	p50 [79], all Rel proteins (GST-pulldown) [78], cRel [80]	p50 [87]	Unknown
**Tissue specific protein expression**	Bone marrow, spleen, lymph nodes, peritoneal lavage, kidney, liver [52]	Heart, skeletal muscle, spleen, kidney, liver, placenta, lung, peripheral blood, leukocytes [73]	Spleen [78]	Brain, lung, kidney, testis, ovary [87]	Human PMBCs [88]
**Knockout phenotype**	Reduction of Peyer’s Patches [53]. Lack of splenic marginal centers [51].	Dermatitis-like skin irritations [74]. Ocular surface inflammation [76]. Resistant to EAE [77].	Less Treg cells [80].	Unknown	Unknown
**Induced via**	LPS [39], IL-9 [54], IL-4 [57]	LPS [61], IL-1 [64], BLP [64], PGN [64], MALP [64], Flagellin [64], peptidoglycan [67], β-glucan [67], CpG-DNA [67], IL-18 [70], IL-12 [70]	LPS [79,82], CD3 [80], anti-IgM [83], CD40 [83]	LPS [87], poly(I:C) [87], CpG-DNA [87], zymosan [87]	LPS [88]
**Direct target genes (ChIP, pulldown, EMSA)**	IP-10 [44], IL-10 [44], MCP-1 [44], CD95 [44], CD40 [44], IL-23p19 [44], CyclinD1 [32], TNFα [39], Gata3 [55]	IL-6 [64], IL8 [72], IL-17 [77], IFNγ [70]	IL-2 [81], IL-6 [82], Foxp3 [80]	Unknown	Unknown

### BCL-3

#### Initial description and structure

BCL-3 was the first identified atypical IκB protein. It consists of an amino-terminal TAD followed by 7 Ankyrin repeats and a second carboxy terminal TAD, displaying an overall length of 448 amino acids (Figure 1). It was first described as a proto-oncogene expressed in patients, which suffered from B-cell chronic lymphocytic leukemia displaying the translocation t (14:19)(q32;q13.1) [30].

#### Function

The oncogenic potential of BCL-3 is illustrated by its capacity to dampen the tumor suppressor p53 and to force Cyclin D1 expression in order to enhance proliferation [31,32]. As the protein is expressed by a variety of different non-Hodgkin and Hodgkin lymphomas it could represent a suitable pharmacological target for the treatment of cancer [33,34]. Electrophoretic mobility shift assays initially revealed that the protein interacts with p50 and p52 and can inhibit DNA binding [32,35]. In contrast to IκBα, which sequesters p50 in the cytoplasm, BCL-3 is localized in the nucleus and alters the subnuclear localization of p50. In COS cells p50 was relatively equally distributed when overexpressed alone, however cotransfection with BCL-3 resulted in its accumulation in nuclear spots [36]. From these analyses it was thought that BCL-3 might act as an anti-repressor by removing suppressive p50/p50 homodimers from the promoters of its target genes, which allows binding of activating p50/p65 or comparable heterodimers and indirectly forces transcriptional activation. Transcriptional repression of BCL-3 is also directly regulated via its binding to HDAC-1, -3 and -6 [37]. In macrophages, LPS is a potent inducer of BCL-3 [38,39], which interacts with p50 to reduce NF-κB-mediated TNF-α production. In agreement with the formation of nuclear suppressor complexes, it was suggested that this effect is mediated via chromatin remodeling. This is based on the fact that HDAC-1 overexpression further enhanced BCL-3-mediated suppression and trichostatin A treatment abrogated the BCL-3-mediated effects [39]. Alternatively, BCL-3 can suppress transcription via block of the ubiquitination of p50 to stabilize a suppressive NF-κB complex within the nucleus [38]. Thus, BCL-3-deficient macrophages display enhanced expression of pro-inflammatory cytokines upon LPS treatment, as degradation of inhibitory p50 homodimers is not blocked. Surprisingly, an early study reported that BCL-3 had the potential to induce transcription directly via an amino terminal proline rich region and a second carboxy-terminal serine/proline rich region [40]. It was later shown that its carboxy-terminal tail enhances transcription via interaction with c-Jun, c-Fos, CREB-binding protein/p300 and the steroid receptor coactivator-1 (SRC-1), with CBP/p300 and SRC-1 having acetyltransferase activity [41]. Remarkably, both the amino and carboxy terminal sequences are needed for the full transcriptional activity of BCL-3, suggesting, that both sequences function cooperatively [40]. An additional part of the nuclear BCL-3 complex is the BCL-3 binding protein B3BP thought to be involved in DNA repair, which forms a complex with p300 and BCL-3 [42]. In addition to p300, the acetyltransferase Tip60 is another interaction partner of BCL-3/p50 complexes, which can enhance transcriptional activity [43]. A recent report nicely demonstrated that p52/BCL-3 complexes bind to A/T and G/C centric NF-κB binding sites sequences, however, with a dramatically altered transcriptional outcome [44]. Via G/C centric elements these complexes induce transcription through Tip60 recruitment, but suppress via binding to A/T centric elements and recruitment of HDAC3.

BCL-3 itself is critically regulated via post-translational modifications, especially via phosphorylation and ubiquitination. It was shown that phosphorylation of BCL-3 via GSK3 regulated BCL-3 degradation and oncogenicity [37,45]. However, its proteasomal degradation in the cytoplasm is regulated by an E3-ligase complex containing TBLR1, which appears to be independently of GSK3 [46]. In all known pathways, NF-κB activity is regulated by several upstream ubiquitination events through the balance between ubiquitin ligases and deubiquitinases [2,47]. CYLD, a K63-deubiquitinase inhibits NF-κB activation in TRAF2-mediated NF-κB signaling pathways [48]. Remarkably, BCL-3 also becomes deubiquitinated by CYLD in the nucleus, upon UV-irradiation. This causes the rapid export of BCL-3 from the nucleus and its inactivation [48].

#### Transgenic mouse models

BCL-3 function was examined using a variety of different transgenic mouse models. Eμ-BCL-3 transgenic mice display splenomegaly, lymphadenopathy and elevated levels of mature B cells in the secondary lymphoid organs, the peritoneal cavity and the bone marrow, suggesting that BCL-3 overexpression renders B cells into a state of hyperactivation [49]. In agreement with this observation, BCL-3-deficient mice display a variety of defects in their humoral immune response. They lack germinal centers in the spleen and show impaired clearance of *Listeria, Streptococci* and *Toxoplasma* infections since they cannot mount a pathogen-specific antibody response [50-52]. Upon *Listeria* infection, reduced IL-12p70 and IFNγ levels were detected, which is presumably the result of increased levels of anti-inflammatory IL-10 produced by macrophages [50]. In addition, like p50-deficient mice, BCL-3-deficient mice display reduced Peyer´s Patches but not a complete absence of them as seen in p52/p100-deficient mice [53]. Besides the role of BCL-3 in B cells, the protein has several properties important for T cells survival and differentiation. In T cells and mast cells, BCL-3 is upregulated by IL-9 and IL-4 via the Jak/STAT pathway [54]. When BCL-3 is absent, induction of GATA-3 by IL-4 is dramatically impaired and, thus, TH2 development [55]. In contrast to this, the generation of IFNγ-producing TH1 cells is not altered in BCL-3 compromised mice [55,56]. However, the protein enhances IFNγ expression in CD8 cells upon second antigen exposure [56]. In addition, IL-4 protects cells from apoptosis via BCL-3. One report demonstrated that BCL-3 expression is lost upon IL-4 deprivation, leading to apoptosis [57]. In agreement, ectopic overexpression of BCL-3 effectively protected cells from IL-4 deprivation-induced death [57]. Consequently, it was suggested that BCL-3 could have anti-apoptotic potential. Indeed, another report demonstrated that BCL-3-deficient T cells are highly sensitive towards activation-induced cell death due to over-activated pro-apoptotic Bim [58]. In line, transgenic overexpression of BCL-3 prolonged T cell survival. In the context of T cells it was further shown, that BCL-3 in cooperation with p52 is important in regulating central tolerance [59]. However, this effect is not intrinsically mediated by T cells, but controlled by medullary thymic epithelial cells, which are required for selection of T cells. These cells display impaired maturation in BCL-3/p100 double-deficient mice, leading to severe autoimmunity [59]. In terms of autoimmune diseases it should be noted, that BCL-3 is also a suppressor of autoimmune diabetes, as BCL-3-deficient NOD mice are more susceptible to autoimmune diabetes and display higher levels of IL-17 [60].

#### Conclusive remarks

The protooncogene BCL-3 displays remarkable versatility in the regulation of NF-κB, for example via NF-κB stabilization in the nucleus or removal of the transription factor from the DNA. Via the recruitment of HAT and HDAC proteins BCL-3 can mediate opposing effects on transcription.

### IκBζ

#### Initial description and structure

IκBζ was first identified by a differential display analysis in a variety of tissues upon i.p. injection of LPS in wildtype mice [61]. It was initially termed “molecule possessing ankyrin repeats induced by LPS” (MAIL), which is still a frequently used name for its murine isoforms [61]. A second study found IκBζ upon IL-1β treatment of OP9 stroma cells leading to its alternative name “interleukin-1 inducible nuclear ankyrin-repeat protein” (INAP) [62]. Up to now, its most common name is IκBζ for both the human and murine proteins [63]. IκBζ consists of a NLS (amino acids 163–178), a transactivation domain (amino acids 329–429), and seven ankyrin repeats (amino acids 450–700) (Figure 1). Early studies demonstrated the nuclear localization of IκBζ in 3T3 and OP9 cells, strong sequence homology of its Ankyrin-repeat containing C-terminal tail to BCL-3 and interaction with p50/p50 homodimers [61,62,64,65]. So far, three murine isoforms of the protein were described. Initially reported were MAIL_L_ (728 amino acids) and the N-terminal truncated isoform MAIL_S_ (629 amino acids) (Figure 1) [61]. Of these two isoforms, the long protein seems to be more prominently expressed [66]. The third isoform, IκBζ-D, is a splicing variant lacking amino acids 236–429 [65]. This deletion results in loss of the suggested transactivation domain (Figure 1). Consequently, IκBζ-D fails to augment NF-κB activity in contrast to the full length protein [65].

#### Function

IκBζ/p50/p50 complexes bind to the IL-6 locus and potentiate transcription in macrophages upon TLR-2, -4 and -9 and IL-1R triggering [64,67]. In agreement, overexpression of the downstream signaling mediators MyD88 and TRAF6 can induce IκBζ mRNA [67]. IκBζ-deficiency causes a reduction of IL-6 and of IL-12p40 expression [64], whereas its overexpression enhances IL-6 production [61]. In contrast to those two cytokines, TNFα transcription is suppressed by IκBζ, which nicely illustrates its dual functionality [65]. So far, several stimuli are known, which force the expression of IκBζ. In addition to the early identified triggers of IκBζ induction, LPS and IL-1β [67], stimulation of macrophages with peptidoglycan, β-glucan and CpG-DNA can also induce IκBζ expression [67]. On the other hand, its mRNA is not detectable upon TNFα or PMA treatment of OP9 cells [62]. Remarkably, the promoter activity of the *Nfkbiz* gene (encoding for IκBζ) upon TNFα treatment is not markedly different compared to stimulation with IL-1β or LPS [68]. IκBζ mRNA is not detectable upon TNFα treatment alone, because it requires stabilization via IL-1β, LPS or IL-17 [68]. TNFα and IL-17 treatment in combination, however, is sufficient to induce IκBζ. Analyses of the murine IκBζ locus also revealed the presence of κB binding sites in its promoter, which suggests its regulation by NF-κB [69]. In agreement with this report, overexpression of dominant negative IκBα can prevent the induction of IκBζ by LPS treatment [67]. Interestingly, ectopic overexpression of the upstream kinases NIK and IKKβ was also sufficient to cause IκBζ-induction in contrast to overexpression of the downstream protein p65 [67].

A recent investigation addressed the function of IκBζ in NK cells. It was shown that IκBζ is induced and recruited to proximal promoter regions of the *ifng* gene upon IL-12 or IL-18 stimulation [70]. As a result of impaired NK cell activation IκBζ-deficient mice were more susceptible to MCMV infections. The effect could be pinpointed to impaired binding of STAT4 to the *ifng* locus. Remarkably, in IκBζ-deficient NK cells STAT4 phosphorylation remained unaffected [70]. Next to the regulation of STAT4, IκBζ was also reported to interact directly with STAT3 via its coiled-coiled domain [71]. Binding of IκBζ results in a dramatic reduction of the transcriptional activity of STAT3. Thereby, transcription of an anti-apoptotic target gene of STAT3, MCL-1, is impaired leading to enhanced apoptosis [71]. Another study revealed its co-localization and interaction with the nuclear fusion oncoprotein FUSS-DDIT3, originating from t(12;16)(q13;p11), which forces the development of myxoid liposarcomas [72]. It was shown that this complex binds to the *IL8* locus and thereby enhances its expression [72]. The modulation of chromatin remodeling through IκBζ was also suggested by the observation that the human protein co-localizes with HDAC-4 and HDAC-5 in nuclear spots [73]. In contrast to the murine protein, human IκBζ presumably interacts with p65 and suppresses its transcriptional activity through HDAC recruitment [73]. However, interaction studies and reporter assays were performed using ectopically overexpressed proteins in HEK 293 cells. As a consequence it still remains uncertain whether murine and human IκBζ show differences regarding the interaction with Rel proteins. It was further shown that the human protein is inducible by IL-1β and TNFα in MCF-7 and Hela cancer cells [73]. In contrast, stimulation of murine macrophages with TNFα alone was not sufficient to induce IκBζ mRNA [62,64,68], indicating that either tumor development alters IκBζ regulation or that the murine and human proteins are regulated in a different fashion. In addition, RNAi-mediated knockdown of IκBζ rendered Hela cells more resistant towards TNFα and CD95-mediated apoptosis [73]. Although certain data indicate differences in the regulation and interaction of murine and human IκBζ, further investigations need to be done to verify functional differences between the proteins of the two species.

#### Transgenic mouse models

IκBζ-deficient mice develop several signs of autoimmune syndromes. These comprise severe skin irritations in the face, neck and periocular regions appearing between weeks 4 and 8 after birth [74]. Further analyses revealed constitutive expression of IκBζ in keratinocytes [75]. Remarkably, its expression was not altered upon LPS treatment *in vivo* or *in vitro*, in contrast to IL-1β treatment, which enhanced IκBζ transcription. This indicates the specific repression of LPS-induced IκBζ expression in keratinocytes. Thus, IκBζ appears to be a mediator of skin homeostasis, whereby its deficiency causes a dermatitis-like phenotype. Remarkably, IκBζ is expressed in a variety of mucosal tissues, such as the ocular surface epithelium [76]. Its deficiency causes chronic inflammation of the ocular surface, leading to infiltration of B220^+^ and CD4^+^ cells in the submucosa. This proposes a role as negative regulator of pathologic progression of ocular surface inflammations [76]. The importance of IκBζ for adaptive immune cells was impressively demonstrated for TH17 cells. IκBζ binds, together with RORγ or RORα, to the *IL-17a* locus [77]. Their combined overexpression could enhance TH17 development from naïve T cells, even without TGFβ and IL-6 treatment. Moreover, IκBζ deficiency impairs TH17 development and results in complete resistance to experimental induced autoimmune encephalomyelitis (EAE) [77]. Thus, IκBζ could be a pharmaceutical target for the treatment of multiple sclerosis (MS).

#### Conclusive remarks

In summary, IκBζ can be considered a pro-inflammatory IκB protein, as it is necessary for the generation of TH17 cells and the production of IL-6 upon LPS exposure. However, as constitutive protein expression in keratinocytes prevent immune cell infiltration in the skin and IκBζ-deficient mice display signs of dermatitis, loss of the protein can also cause inflammatory syndromes.

### IκB_NS_

#### Initial description and structure

IκB_NS_, also known as TA-NFKBH and Nfkbid, consists of 327 amino acids and, therefore, is the smallest member of the BCL-3 subfamily [78]. IκB_NS_ was initially identified by RDA analysis, investigating genes induced upon negative selection of T cells in the thymus [78]. It consists almost entirely of six ankyrin repeats and short C- and N-terminal tails, but no transactivation domains were reported yet (Figure 1). The interaction of IκB_NS_ with other NF-κB family members is not entirely clear. It was shown that overexpressed IκB_NS_ predominantly interacts with p50 but not p65 in RAW264.7 macrophages [79]. However, pulldown experiments using GST-IκB_NS_ and protein extracts from stimulated N15 TCR transgenic thymocytes demonstrated binding to cytoplasmic and nuclear p50 as well as nuclear p52, p65, RelB and c-Rel [78]. Therefore, it is conceivable that IκB_NS_ can interact with several different NF-κB dimers in the nucleus. One study reported mild interaction of endogenous IκB_NS_ and c-Rel in stimulated T cells [80]. The presence of a specific interaction might depend on posttranslational modifications and on the analyzed cell type.

#### Transgenic mouse models and function

The generation of IκB_NS_-deficient mice revealed that the protein is dispensable for negative selection, since CD4 and CD8 T cell numbers and Vβ expression are identical between IκB_NS_-deficient and wildtype mice [81]. Moreover, analyses of TCR specificities indicated unaltered reactivity to antigens compared to wildtype mice. However, it was shown that IκB_NS_ is inducible in mature CD4 T cells upon TCR stimulation [80]. Its deficiency causes reduced expression of IL-2 and IFNγ upon stimulation by anti-CD3 and anti-CD28 and mildly impaired proliferation, which could be overcome by treatment with PMA and ionomycin [81]. In IκB_NS_-deficient macrophages and DCs, however, LPS triggering resulted in prolonged and enhanced expression of IL-6 and IL-12p40 [79,82]. To this end it is thought that a complex containing p50 and IκB_NS_ is required to terminate IL-6 expression. The reductions of IL-6 and IL-12p40 on the one hand and the inductions of IL-2 and IFNγ on the other hand underline the dual function of atypical IκB proteins as repressors or inducers of transcription also for IκB_NS_. It is also interesting, that IκB_NS_ acts antagonistic to IκBζ in the regulation of IL-6 in macrophages [64,79]. Next to macrophages and T cells, a recent report suggested a role for IκB_NS_ in B cell development, as it is induced by LPS, anti-IgM and CD40 triggering [83]. Notably, IκB_NS_-deficient mice lack the entire B1 B cell compartment and display reduced B cell numbers in the marginal zone [83,84]. Corresponding to impaired T cell proliferation upon TCR triggering, proliferation was reduced upon LPS and anti-CD40 triggering in IκB_NS_-deficient B cells [83]. In agreement with the impaired generation of plasma cells *in vitro*, serum IgM and IgG3 levels were dramatically reduced and less antigen-specific antibodies were produced upon *influenza* infection of IκB_NS_-deficient mice. Remarkably, one report demonstrated, that IκB_NS_ expression is suppressed by an AP-1/Foxp3 complex [85]. Foxp3 governs the generation and function of immunosuppressive regulatory T cells. Of note, IL-2 secretion in Treg cells is prevented. Thus, it is conceivable that IκB_NS_ repression might ensure silencing of IL-2 transcription in Treg cells, as it is needed for IL-2 induction upon activation of CD4 and CD8 cells [81]. Although the protein is repressed in Foxp3^+^ Tregs, IκB_NS_ is important for the maturation of Foxp3^-^ Treg precursors [80]. Thus, Treg cells are reduced in IκB_NS_-deficient mice. Whether or not human IκB_NS_ functions in a similar fashion remains unknown. The sole report on human IκB_NS_ demonstrated that its mRNA is induced upon IL-1β treatment of immortalized human gingival fibroblasts, along with the other NF-κB proteins p50, p52, p65, RelB IκBα, IκBϵ, and IκBζ [86].

#### Conclusive remarks

IκB_NS_ is necessary for the generation of immunosuppressive Treg cells and the termination of pro-inflammatory cytokines like IL-6 and IL12p40. On the other hand it promotes germinal center reactions and IL-2 induction. Thus, the protein mediates immune activation as well as suppression. Therefore, it is an important regulator of immune homeostasis, although it cannot simply be classified as a pro- nor anti-inflammatory signaling protein.

### IκBη

IκBη is the most recently identified member of the BCL-3 subfamily, found by microarray analyses of bone marrow derived DCs [87]. It was shown that the protein made up of 516 amino acids is induced upon LPS, polyI:C, CpG DNA and zymosan treatment in RAW264.7 macrophages [87]. In contrast to the other BCL-3 proteins, it consists of 8 ankyrin domains and a prolonged carboxy terminal tail (Figure 1). Co-Immunoprecipitation experiments demonstrated its interaction with p50, but not with p65. Its siRNA-mediated knockdown led to the loss of the expression of several pro-inflammatory genes, such as the classical NF-κB target genes *Il6*, *Il1b* and *ifnb*[87]. In agreement with the reduced expression of cytokines upon IκBη loss, its overexpression mediated increased luciferase activity of NF-κB consensus constructs [87]. The obvious functional similarity to IκBζ suggests redundancy of the two proteins, but the prolonged carboxy terminal tail is unique to IκBη (Figure 1). Generation of IκBη-deficient mice is essential to further determine functional differences or redundancies between the two proteins.

### IκBL

It is still a matter of debate, whether the two reported IκBL isoforms, α(L) and α(S), belong to the group of IκB proteins, because no REL protein was identified as an interaction partner so far. Nevertheless, the protein contains ankyrin repeats, is localized in the nucleus and suppresses NF-κB target genes TNFα and IL-6 [88]. Furthermore, fluorescent microscopy revealed its localisation in nuclear dot-like structures [89], a property of BCL-3 [36], IκBζ [73], IκBη [87] as well as IκB_NS_ (unpublished data). Although both reports strongly indicate the identification of an additional nuclear IκB protein, interaction with an NF-κB subunit is a prerequisite to consider IκBL part of this class.

## Conclusions

The BCL-3 subfamily of IκB proteins alters NF-κB activity in a positive or negative fashion. BCL-3, IκBζ, IκB_NS_ and IκBη exhibit their function in the nucleus, via association with NF-κB subunits on the DNA. Their main interaction partners are p50 and p52 within the NF-κB pathway [32,36,62,78,87]. The observed interaction of overexpressed human IκBζ with p65 and interaction studies using GST-IκB_NS_ and *in vitro* translated REL proteins suggest, that atypical IκB proteins can also bind to the other NF-κB subunits [73,78]. However, these interactions might be cell type specific and could depend on specific stimuli or posttranslational modifications of IκBs and REL proteins as well. Atypical IκBs exert their transcriptional function by a magnitude of mechanisms, whose regulations and interplay are not completely understood. BCL-3 stabilizes p50 homodimers on the DNA to silence specific genes because their capacity to compete with activating p50/p65 heterodimers is increased. On the other hand, it is also possible that BCL-3 removes p50 homodimers and relocalizes them to the nucleus in dot-like structures that are associated with HDAC proteins in order to repress transcription [37]. In both examples, BCL-3 acts as a factor, which regulates the maintenance of NF-κB binding to the DNA. Remarkably, atypical IκBs can also recruit proteins, which alter transcription via changes of the chromatin structure. BCL-3 interaction with the histone acetyl transferases p300 and Tip60 [42,43], as well as co-localization of IκBζ with HDAC4 and HDAC5 was observed using confocal microscopy [73]. Apparently the interaction with chromatin remodeling enzymes is a dynamic process, as BCL-3 does not exclusively bind to acetyl transferases, but can also co-localize with HDAC proteins in the nucleus. Further analyses of IκB_NS_ and IκBη are needed to determine, whether recruitment of histone modifying enzymes is a mechanism common to all atypical IκB proteins.

**Figure 2:**
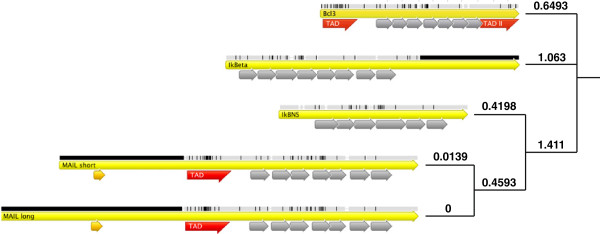
**Homology of atypical IκB proteins.** Homology tree between the atypical IκB proteins IκBζ long, IκBζ short, IκB_NS_, IκBη and BCL-3 is shown. Numbers indicate evolutionary distance between the different proteins. Tree was generated by Geneious v5.3 software (Drummond et al., 2010; available at www.geneious.com/).

Remarkably, all atypical IκBs are induced via LPS stimulation (Table 1) [39,61,79,87]. Although the sequence similarities of the proteins is high (Figure 2), it remains unknown, how and if atypical IκB proteins cooperate or compete with each other during the regulation of common target genes and common interaction partners like p50. As an example, IκBη and IκBζ were both shown to force IL-6 production in macrophages, as loss of these proteins shortened the expression period and the level of the secreted cytokines [64,87]. These data suggest a cooperative function. Nevertheless, it is unknown, whether this depends on direct protein interaction between the two IκBs or whether it is mediated via two different κB binding sites, or via two different dimers, which are sequentially exchanged. On the other hand, IL-6 expression is repressed by IκB_NS_, since its loss prolongs the period of cytokine secretion and increases their expression level [79,82]. Thus, IκB_NS_ acts in an opposite fashion to IκBζ and IκBη. It is highly likely, that these proteins are sequentially recruited to the IL-6 locus, to regulate the induction and termination of cytokine expression. However, comprehensive studies are needed, to verify this hypothesis.

Several reports demonstrated the oncogenic potential of BCL-3, as the protein is highly upregulated in a variety of cancer cells, suppresses the activity of p53 and acts in an anti-apoptotic fashion [31-33]. Thus, the analyses of the BCL-3 expression status might be suitable for determining the prognosis of tumor progression and the disease course. It might also represent a suitable pharmacological target for cancer treatment. Atypical IκBs are of particular importance for the development of distinct T helper cell subsets. BCL-3 is an essential mediator of TH2 development via GATA-3 upregulation and IL-4 secretion, without affecting the TH1 subset [55]. IκBζ-deficient mice are completely protected from EAE as IκBζ-deficient T cells fail to develop into IL-17 producing TH17 cells [77]. At last, IκB_NS_ drives the development of regulatory T cells by binding to regulatory elements of the Foxp3 locus, whereby IκB_NS_-deficient mice display reduced Treg numbers [80]. Thus, there exists compelling evidence that atypical IκBs are specific regulators of T helper cell subsets. Therefore, pharmacological targeting of atypical IκBs might help to develop therapies to treat diseases, which depend on a certain T cell subset. As atypical IκBs are involved in a variety of cellular processes, but the understanding of their molecular regulation and relationship remains incomplete, further studies are necessary to uncover their pharmacological potential in the future.

## Abbreviations

AP-1: Activator protein 1; BCL-3: B-cell lymphoma 3-encoded protein; CD: Cluster of differentiation; ChIP: Chromatin Immunoprecipitation; CREB: cAMP response element-binding protein; CtBP: C-terminal-binding protein 1; CYLD: Cylindromatosis; DNA: Deoxyribonucleic acid; EAE: Experimantal induced autoimmune encephalomyelitis; Foxp3: Forkhead box protein 3; GSK3: Glycogen synthase kinase 3; GST: Glutathione S-transferase; HAT: Histone acetyl transferase; HDAC: Histone deacetylase; IFNγ: Interferon gamma; Ig: Immunoglobuline; IκB: Inhibtior of NF-κB; IKK: IκB Kinase; IL: Interleukin; INAP: Interleukin-1 inducible nuclear ankyrin-repeat protein; JAK: Janus kinase; LPS: Lipopolysaccharide; LSD1: Lysine-specific demethylase 1; MAIL: Molecule possessing ankyrin repeats induced by LPS; MCMV: Murine cytomegaly virus; MS: Multiple Sclerosis; MyD88: Myeloid differentiation primary response gene (88); NCOR: Nuclear receptor co-repressor; NEMO: NF-κB essential modulator; NF-κB: Nuclear factor kappa B; NIK: NF-κB inducing kinase; NK: Natural killer; NLS: Nuclear localization signal; NOD: Non-obese diabetic; RDA: Representational difference analysis; RHD: REL homology domain; RNA: Ribonucleic acid; RAR: Retinoic acid receptor; ROR: RAR-related orphan receptor; SRC-1: Steroid receptor coactivator 1; STAT: Signal transducer and activator of transcription; TAD: Transactivation domain; TNFα: Tumor necrosis factor alpha; TRAF2: TNF receptor associated factor.

## Competing interests

The authors declare that they have no competing interests.

## Authors’ contributions

All authors contributed in the conception and writing of the manuscript. All authors edited and approved the final version.

## References

[B1] Baeuerle PA Baltimore D A 65-kappaD subunit of active NF-kappaB is required for inhibition of NF-kappaB by I kappaB Genes Dev 1989 3 11 1689 1698 10.1101/gad.3.11.1689 2691328

[B2] Hayden MS Ghosh S NF-kappaB, the first quarter-century: remarkable progress and outstanding questions Genes Dev 2012 26 3 203 234 10.1101/gad.183434.111 22302935 PMC3278889

[B3] Chen LF Greene WC Shaping the nuclear action of NF-kappaB Nat Rev Mol Cell Biol 2004 5 5 392 401 10.1038/nrm1368 15122352

[B4] Siggers T Principles of dimer-specific gene regulation revealed by a comprehensive characterization of NF-kappaB family DNA binding Nat Immunol 2012 13 1 95 102 22101729 10.1038/ni.2151 PMC3242931

[B5] Wang JK Evaluating the binding affinities of NF-kappaB p50 homodimer to the wild-type and single-nucleotide mutant Ig-kappaB sites by the unimolecular dsDNA microarray Anal Biochem 2003 316 2 192 201 10.1016/S0003-2697(03)00049-6 12711340

[B6] Wong D Extensive characterization of NF-kappaB binding uncovers non-canonical motifs and advances the interpretation of genetic functional traits Genome Biol 2011 12 7 R70 10.1186/gb-2011-12-7-r70 21801342 PMC3218832

[B7] Razani B Reichardt AD Cheng G Non-canonical NF-kappaB signaling activation and regulation: principles and perspectives Immunol Rev 2011 244 1 44 54 10.1111/j.1600-065X.2011.01059.x 22017430

[B8] Rothwarf DM IKK-gamma is an essential regulatory subunit of the IkappaB kinase complex Nature 1998 395 6699 297 300 10.1038/26261 9751060

[B9] Zandi E The IkappaB kinase complex (IKK) contains two kinase subunits, IKKalpha and IKKbeta, necessary for IkappaB phosphorylation and NF-kappaB activation Cell 1997 91 2 243 252 10.1016/S0092-8674(00)80406-7 9346241

[B10] Baeuerle PA Baltimore D I kappa B: a specific inhibitor of the NF-kappa B transcription factor Science 1988 242 4878 540 546 10.1126/science.3140380 3140380

[B11] Baeuerle PA Baltimore D Activation of DNA-binding activity in an apparently cytoplasmic precursor of the NF-kappa B transcription factor Cell 1988 53 2 211 217 10.1016/0092-8674(88)90382-0 3129195

[B12] Davis N Rel-associated pp 40: an inhibitor of the rel family of transcription factors Science 1991 253 5025 1268 1271 10.1126/science.1891714 1891714

[B13] Haskill S Characterization of an immediate-early gene induced in adherent monocytes that encodes I kappa B-like activity Cell 1991 65 7 1281 1289 10.1016/0092-8674(91)90022-Q 1829648

[B14] Huxford T The crystal structure of the IkappaBalpha/NF-kappaB complex reveals mechanisms of NF-kappaB inactivation Cell 1998 95 6 759 770 10.1016/S0092-8674(00)81699-2 9865694

[B15] Malek S X-ray crystal structure of an IkappaBbeta x NF-kappaB p65 homodimer complex J Biol Chem 2003 278 25 23094 23100 10.1074/jbc.M301022200 12686541

[B16] Baeuerle PA IkappaB-NF-kappaB structures: at the interface of inflammation control Cell 1998 95 6 729 731 10.1016/S0092-8674(00)81694-3 9865689

[B17] Henkel T Rapid proteolysis of I kappa B-alpha is necessary for activation of transcription factor NF-kappa B Nature 1993 365 6442 182 185 10.1038/365182a0 8371761

[B18] Mellits KH Hay RT Goodbourn S Proteolytic degradation of MAD3 (I kappa B alpha) and enhanced processing of the NF-kappa B precursor p105 are obligatory steps in the activation of NF-kappa B Nucleic Acids Res 1993 21 22 5059 5066 10.1093/nar/21.22.5059 8255759 PMC310617

[B19] Regnier CH Identification and characterization of an IkappaB kinase Cell 1997 90 2 373 383 10.1016/S0092-8674(00)80344-X 9244310

[B20] DiDonato JA A cytokine-responsive IkappaB kinase that activates the transcription factor NF-kappaB Nature 1997 388 6642 548 554 10.1038/41493 9252186

[B21] Chen Z Signal-induced site-specific phosphorylation targets I kappa B alpha to the ubiquitin-proteasome pathway Genes Dev 1995 9 13 1586 1597 10.1101/gad.9.13.1586 7628694

[B22] Yaron A Inhibition of NF-kappa-B cellular function via specific targeting of the I-kappa-B-ubiquitin ligase EMBO J 1997 16 21 6486 6494 10.1093/emboj/16.21.6486 9351830 PMC1170254

[B23] Winston JT The SCFbeta-TRCP-ubiquitin ligase complex associates specifically with phosphorylated destruction motifs in IkappaBalpha and beta-catenin and stimulates IkappaBalpha ubiquitination in vitro Genes Dev 1999 13 3 270 283 10.1101/gad.13.3.270 9990852 PMC316433

[B24] Coope HJ CD40 regulates the processing of NF-kappaB2 p100 to p52 EMBO J 2002 21 20 5375 5385 10.1093/emboj/cdf542 12374738 PMC129074

[B25] Senftleben U Activation by IKKalpha of a second, evolutionary conserved, NF-kappa B signaling pathway Science 2001 293 5534 1495 1499 10.1126/science.1062677 11520989

[B26] Solan NJ RelB cellular regulation and transcriptional activity are regulated by p100 J Biol Chem 2002 277 2 1405 1418 10.1074/jbc.M109619200 11687592

[B27] Neri A B cell lymphoma-associated chromosomal translocation involves candidate oncogene lyt-10, homologous to NF-kappa B p50 Cell 1991 67 6 1075 1087 10.1016/0092-8674(91)90285-7 1760839

[B28] Fong A Sun SC Genetic evidence for the essential role of beta-transducin repeat-containing protein in the inducible processing of NF-kappa B2/p100 J Biol Chem 2002 277 25 22111 22114 10.1074/jbc.C200151200 11994270

[B29] Mankan AK NF-kappaB regulation: the nuclear response J Cell Mol Med 2009 13 4 631 643 10.1111/j.1582-4934.2009.00632.x 19438970 PMC3822870

[B30] Ohno H Takimoto G McKeithan TW The candidate proto-oncogene bcl-3 is related to genes implicated in cell lineage determination and cell cycle control Cell 1990 60 6 991 997 10.1016/0092-8674(90)90347-H 2180580

[B31] Kashatus D Cogswell P Baldwin AS Expression of the Bcl-3 proto-oncogene suppresses p53 activation Genes Dev 2006 20 2 225 235 10.1101/gad.1352206 16384933 PMC1356113

[B32] Park SG Up-regulation of cyclin D1 by HBx is mediated by NF-kappaB2/BCL3 complex through kappaB site of cyclin D1 promoter J Biol Chem 2006 281 42 31770 31777 10.1074/jbc.M603194200 16940298

[B33] Canoz O Immunohistochemical detection of BCL-3 in lymphoid neoplasms: a survey of 353 cases Mod Pathol 2004 17 8 911 917 10.1038/modpathol.3800140 15105810

[B34] Mathas S Elevated NF-kappaB p50 complex formation and Bcl-3 expression in classical Hodgkin, anaplastic large-cell, and other peripheral T-cell lymphomas Blood 2005 106 13 4287 4293 10.1182/blood-2004-09-3620 16123212

[B35] Hatada EN The ankyrin repeat domains of the NF-kappa B precursor p105 and the protooncogene bcl-3 act as specific inhibitors of NF-kappa B DNA binding Proc Natl Acad Sci USA 1992 89 6 2489 2493 10.1073/pnas.89.6.2489 1532257 PMC48684

[B36] Zhang Q BCL3 encodes a nuclear protein which can alter the subcellular location of NF-kappa B proteins Mol Cell Biol 1994 14 6 3915 3926 8196632 10.1128/mcb.14.6.3915-3926.1994 PMC358758

[B37] Viatour P GSK3-mediated BCL-3 phosphorylation modulates its degradation and its oncogenicity Mol Cell 2004 16 1 35 45 10.1016/j.molcel.2004.09.004 15469820

[B38] Carmody RJ Negative regulation of toll-like receptor signaling by NF-kappaB p50 ubiquitination blockade Science 2007 317 5838 675 678 10.1126/science.1142953 17673665

[B39] Wessells J BCL-3 and NF-kappaB p50 attenuate lipopolysaccharide-induced inflammatory responses in macrophages J Biol Chem 2004 279 48 49995 50003 10.1074/jbc.M404246200 15465827

[B40] Bours V The oncoprotein Bcl-3 directly transactivates through kappa B motifs via association with DNA-binding p50B homodimers Cell 1993 72 5 729 739 10.1016/0092-8674(93)90401-B 8453667

[B41] Na SY Bcl3, an IkappaB protein, stimulates activating protein-1 transactivation and cellular proliferation J Biol Chem 1999 274 40 28491 28496 10.1074/jbc.274.40.28491 10497212

[B42] Watanabe N Wachi S Fujita T Identification and characterization of BCL-3-binding protein: implications for transcription and DNA repair or recombination J Biol Chem 2003 278 28 26102 26110 10.1074/jbc.M303518200 12730195

[B43] Dechend R The Bcl-3 oncoprotein acts as a bridging factor between NF-kappaB/Rel and nuclear co-regulators Oncogene 1999 18 22 3316 3323 10.1038/sj.onc.1202717 10362352

[B44] Wang VY The transcriptional specificity of NF-kappaB dimers is coded within the kappaB DNA response elements Cell Rep 2012 2 4 824 839 10.1016/j.celrep.2012.08.042 23063365 PMC4167904

[B45] Viatour P Protein phosphorylation as a key mechanism for the regulation of BCL-3 activity Cell Cycle 2004 3 12 1498 1501 10.4161/cc.3.12.1328 15611665

[B46] Keutgens A The repressing function of the oncoprotein BCL-3 requires CtBP, while its polyubiquitination and degradation involve the E3 ligase TBLR1 Mol Cell Biol 2010 30 16 4006 4021 10.1128/MCB.01600-09 20547759 PMC2916444

[B47] Wei N Serino G Deng XW The COP9 signalosome: more than a protease Trends Biochem Sci 2008 33 12 592 600 10.1016/j.tibs.2008.09.004 18926707

[B48] Massoumi R Cyld inhibits tumor cell proliferation by blocking Bcl-3-dependent NF-kappaB signaling Cell 2006 125 4 665 677 10.1016/j.cell.2006.03.041 16713561

[B49] Ong ST Lymphadenopathy, splenomegaly, and altered immunoglobulin production in BCL3 transgenic mice Oncogene 1998 16 18 2333 2343 10.1038/sj.onc.1201771 9620550

[B50] Riemann M The IkappaB protein Bcl-3 negatively regulates transcription of the IL-10 gene in macrophages J Immunol 2005 175 6 3560 3568 16148099 10.4049/jimmunol.175.6.3560

[B51] Schwarz EM Immunological defects in mice with a targeted disruption in Bcl-3 Genes Dev 1997 11 2 187 197 10.1101/gad.11.2.187 9009202

[B52] Franzoso G Critical roles for the Bcl-3 oncoprotein in T cell-mediated immunity, splenic microarchitecture, and germinal center reactions Immunity 1997 6 4 479 490 10.1016/S1074-7613(00)80291-5 9133427

[B53] Paxian S Abnormal organogenesis of Peyer's patches in mice deficient for NF-kappaB1, NF-kappaB2, and Bcl-3 Gastroenterology 2002 122 7 1853 1868 10.1053/gast.2002.33651 12055593

[B54] Richard M Interleukin-9 regulates NF-kappaB activity through BCL3 gene induction Blood 1999 93 12 4318 4327 10361130

[B55] Corn RA Opposing roles for RelB and Bcl-3 in regulation of T-box expressed in T cells, GATA-3, and Th effector differentiation J Immunol 2005 175 4 2102 2110 16081776 10.4049/jimmunol.175.4.2102

[B56] Chilton PM Mitchell TC CD8 T cells require Bcl-3 for maximal gamma interferon production upon secondary exposure to antigen Infect Immun 2006 74 7 4180 4189 10.1128/IAI.01749-05 16790793 PMC1489710

[B57] Rebollo A Bcl-3 expression promotes cell survival following interleukin-4 deprivation and is controlled by AP1 and AP1-like transcription factors Mol Cell Biol 2000 20 10 3407 3416 10.1128/MCB.20.10.3407-3416.2000 10779330 PMC85633

[B58] Bauer A The NF-kappaB regulator Bcl-3 and the BH3-only proteins Bim and Puma control the death of activated T cells Proc Natl Acad Sci USA 2006 103 29 10979 10984 10.1073/pnas.0603625103 16832056 PMC1544160

[B59] Zhang X A role for the IkappaB family member Bcl-3 in the control of central immunologic tolerance Immunity 2007 27 3 438 452 10.1016/j.immuni.2007.07.017 17869136 PMC2000815

[B60] Ruan Q Roles of Bcl-3 in the pathogenesis of murine type 1 diabetes Diabetes 2010 59 10 2549 2557 10.2337/db10-0480 20622172 PMC3279524

[B61] Kitamura H MAIL, a novel nuclear I kappa B protein that potentiates LPS-induced IL-6 production FEBS Lett 2000 485 1 53 56 10.1016/S0014-5793(00)02185-2 11086164

[B62] Haruta H Kato A Todokoro K Isolation of a novel interleukin-1-inducible nuclear protein bearing ankyrin-repeat motifs J Biol Chem 2001 276 16 12485 12488 10.1074/jbc.C100075200 11278262

[B63] Yamazaki S Muta T Takeshige K A novel IkappaB protein, IkappaB-zeta, induced by proinflammatory stimuli, negatively regulates nuclear factor-kappaB in the nuclei J Biol Chem 2001 276 29 27657 27662 10.1074/jbc.M103426200 11356851

[B64] Yamamoto M Regulation of Toll/IL-1-receptor-mediated gene expression by the inducible nuclear protein IkappaBzeta Nature 2004 430 6996 218 222 10.1038/nature02738 15241416

[B65] Motoyama M Positive and negative regulation of nuclear factor-kappaB-mediated transcription by IkappaB-zeta, an inducible nuclear protein J Biol Chem 2005 280 9 7444 7451 15618216 10.1074/jbc.M412738200

[B66] Kitamura H Bacterial lipopolysaccharide-induced expression of the IkappaB protein MAIL in B-lymphocytes and macrophages Arch Histol Cytol 2003 66 1 53 62 10.1679/aohc.66.53 12703554

[B67] Eto A Essential roles for NF-kappa B and a Toll/IL-1 receptor domain-specific signal(s) in the induction of I kappa B-zeta Biochem Biophys Res Commun 2003 301 2 495 501 10.1016/S0006-291X(02)03082-6 12565889

[B68] Yamazaki S Stimulus-specific induction of a novel nuclear factor-kappaB regulator, IkappaB-zeta, via Toll/Interleukin-1 receptor is mediated by mRNA stabilization J Biol Chem 2005 280 2 1678 1687 15522867 10.1074/jbc.M409983200

[B69] Shiina T Genomic organization, chromosomal localization, and promoter analysis of the mouse Mail gene Immunogenetics 2001 53 8 649 655 10.1007/s00251-001-0376-x 11797098

[B70] Miyake T IkappaBzeta is essential for natural killer cell activation in response to IL-12 and IL-18 Proc Natl Acad Sci USA 2010 107 41 17680 17685 10.1073/pnas.1012977107 20876105 PMC2955119

[B71] Wu Z Nuclear protein IkappaB-zeta inhibits the activity of STAT3 Biochem Biophys Res Commun 2009 387 2 348 352 10.1016/j.bbrc.2009.07.023 19595668

[B72] Goransson M The myxoid liposarcoma FUS-DDIT3 fusion oncoprotein deregulates NF-kappaB target genes by interaction with NFKBIZ Oncogene 2009 28 2 270 278 10.1038/onc.2008.378 18850010

[B73] Totzke G A novel member of the IkappaB family, human IkappaB-zeta, inhibits transactivation of p65 and its DNA binding J Biol Chem 2006 281 18 12645 12654 10.1074/jbc.M511956200 16513645

[B74] Shiina T Targeted disruption of MAIL, a nuclear IkappaB protein, leads to severe atopic dermatitis-like disease J Biol Chem 2004 279 53 55493 55498 10.1074/jbc.M409770200 15491998

[B75] Oonuma T Role of NF-kappaB in constitutive expression of MAIL in epidermal keratinocytes J Vet Med Sci 2007 69 3 279 284 10.1292/jvms.69.279 17409644

[B76] Ueta M Spontaneous ocular surface inflammation and goblet cell disappearance in I kappa B zeta gene-disrupted mice Invest Ophthalmol Vis Sci 2005 46 2 579 588 10.1167/iovs.04-1055 15671285

[B77] Okamoto K IkappaBzeta regulates T(H)17 development by cooperating with ROR nuclear receptors Nature 2010 464 7293 1381 1385 10.1038/nature08922 20383124

[B78] Fiorini E Peptide-induced negative selection of thymocytes activates transcription of an NF-kappa B inhibitor Mol Cell 2002 9 3 637 648 10.1016/S1097-2765(02)00469-0 11931770

[B79] Hirotani T The nuclear IkappaB protein IkappaBNS selectively inhibits lipopolysaccharide-induced IL-6 production in macrophages of the colonic lamina propria J Immunol 2005 174 6 3650 3657 15749903 10.4049/jimmunol.174.6.3650

[B80] Schuster M IkappaB(NS) Protein Mediates Regulatory T Cell Development via Induction of the Foxp3 Transcription Factor Immunity 2012 9 3 426 428 10.1016/j.immuni.2012.08.023 23200824

[B81] Touma M Functional role for I kappa BNS in T cell cytokine regulation as revealed by targeted gene disruption J Immunol 2007 179 3 1681 1692 17641034 10.4049/jimmunol.179.3.1681

[B82] Kuwata H IkappaBNS inhibits induction of a subset of Toll-like receptor-dependent genes and limits inflammation Immunity 2006 24 1 41 51 10.1016/j.immuni.2005.11.004 16413922

[B83] Touma M Impaired B cell development and function in the absence of IkappaBNS J Immunol 2011 187 8 3942 3952 10.4049/jimmunol.1002109 21900180 PMC3348541

[B84] Arnold CN A forward genetic screen reveals roles for Nfkbid, Zeb1, and Ruvbl2 in humoral immunity Proc Natl Acad Sci USA 2012 109 31 12286 12293 10.1073/pnas.1209134109 22761313 PMC3411946

[B85] Marson A Foxp3 occupancy and regulation of key target genes during T-cell stimulation Nature 2007 445 7130 931 935 10.1038/nature05478 17237765 PMC3008159

[B86] Vardar-Sengul S Expression profile of human gingival fibroblasts induced by interleukin-1beta reveals central role of nuclear factor-kappa B in stabilizing human gingival fibroblasts during inflammation J Periodontol 2009 80 5 833 849 10.1902/jop.2009.080483 19405838 PMC4150685

[B87] Yamauchi S Ito H Miyajima A IkappaBeta, a nuclear IkappaB protein, positively regulates the NF-kappaB-mediated expression of proinflammatory cytokines Proc Natl Acad Sci USA 2010 107 26 11924 11929 10.1073/pnas.0913179107 20547855 PMC2900662

[B88] Chiba T IkappaBL, a novel member of the nuclear IkappaB family, inhibits inflammatory cytokine expression FEBS Lett 2011 585 22 3577 3581 10.1016/j.febslet.2011.10.024 22024480

[B89] Atzei P Cactin targets the MHC class III protein IkappaB-like (IkappaBL) and inhibits NF-kappaB and interferon-regulatory factor signaling pathways J Biol Chem 2010 285 47 36804 36817 10.1074/jbc.M110.139113 20829348 PMC2978609

